# Developing a realist theory of psychosocial rehabilitation: the Clubhouse model

**DOI:** 10.1186/s12913-018-3265-9

**Published:** 2018-06-13

**Authors:** Christina Mutschler, Jen Rouse, Kelly McShane, Criss Habal-Brosek

**Affiliations:** 1 Department of Psychology, Ryerson University, 350 Victoria Street, Toronto, ON M5B 2K3 Canada; 2 Progress Place, 576 Church Street, Toronto, ON M4Y 2E3 Canada

**Keywords:** Psychosocial rehabilitation, Clubhouse model, Realist evaluation, Severe mental illness

## Abstract

**Background:**

Psychosocial rehabilitation is a service that supports recovery from mental illness by providing opportunities for skill development, self-determination, and social interaction. One type of psychosocial rehabilitation is the Clubhouse model. The purpose of the current project was to create, test, and refine a realist theory of psychosocial rehabilitation at Progress Place, an accredited Clubhouse.

**Method:**

Realist evaluation is a theory driven evaluation that uncovers contexts, mechanisms, and outcomes, in order to develop a theory as to how a program works. The current study involved two phases, encompassing four steps: Phase 1 included (1) initial theory development and (2) initial theory refinement; and Phase 2 included (3) theory testing and (4) refinement.

**Results:**

The data from this two-phase approach identified three demi-regularities of recovery comprised of specific mechanisms and outcomes: the Restorative demi-regularity, the Reaffirming demi-regularity, and the Re-engaging demi-regularity. The theory derived from these demi-regularities suggests that there are various mechanisms that produce outcomes of recovery from the psychosocial rehabilitation perspective, and as such, it is necessary that programs promote a multifaceted, holistic perspective on recovery.

**Conclusions:**

The realist evaluation identified that Progress Place promotes recovery for members. Additional research on the Clubhouse model should be conducted to further validate that the model initiates change and promotes recovery outcomes.

**Electronic supplementary material:**

The online version of this article (10.1186/s12913-018-3265-9) contains supplementary material, which is available to authorized users.

## Background

Psychosocial rehabilitation is type of service for supporting mental illness recovery that contrasts with standard acute care in that it assumes that each person is capable of improving their own functioning. In a psychosocial rehabilitation center, service recipients are motivated towards better use of their social, emotional, mental, and working capacity. The approach focuses on the strengths and abilities that will allow for independent functioning and fulfillment of societal roles, and assumes that growth and recovery is a process that continuously occurs throughout each individual’s life [1].

One specific type of psychosocial rehabilitation is the Clubhouse model. Clubhouses provide individuals experiencing mental illness services and opportunities to live meaningfully within their communities [2]. Clubhouses define their service users as members, rather than patients or clients, because they actively engage in all aspects of the Clubhouse, in contrast to passive service users. Clubhouse members are afforded self-determination, in that they are given the choice of how often they would like to attend, what kind of programming they would like to participate in, and which staff with whom they would like to work. Clubhouses provide work experiences, social events, and housing, as well as encouraging community participation in order to help members develop and maintain healthy lifestyles [2]. Clubhouses aim to strengthen and increase the social networks of individuals living with severe mental illness by providing an environment that promotes a sense of community and belonging through peer support. Research suggests that Clubhouse members experience reduced symptoms of mental illness and enhanced recovery outcomes [3–7].

Progress Place is an accredited Clubhouse located in Toronto, Ontario that recently underwent a participatory evaluation in order to identify mechanisms that lead to recovery outcomes for individuals with mental illness [8]. This evaluation allowed for the identification of mechanisms and outcomes, providing the groundwork for further research to identify an underlying theory of how these mechanisms and outcomes occur together. Therefore, the present study used a realist perspective to combine mechanisms and outcomes in order to develop and test a theory of psychosocial rehabilitation.

### Theoretical approach

Previous research on Clubhouses has identified positive outcomes; however, the Clubhouse model has never been evaluated from a realist perspective as to how the principles guiding the model work to achieve outcomes. A realist evaluation offers the opportunity to create a program theory by asking the question, what works, for whom, and under what circumstances [9]. The theory-based evaluation involves the development and refinement of a program theory, which includes contexts, mechanisms, and outcomes, or “CMO” configurations. From the realist perspective, contextual variables either allow for or restrict the development of causal mechanisms and outcome variables. The development of a realist theory provides answers to how mechanisms and outcomes are constrained by individual, program-specific, or societal contexts [10]. Mechanism variables are defined as the cognitive or affective responses of those who are using the program [10]. Mechanisms provide insight into how outcomes are achieved, rather than traditional systematic reviews that merely focus on whether or not the outcomes were achieved. A previous study has identified various mechanisms and outcomes at Progress Place [8], although a knowledge gap remains in understanding the underlying theory of how these mechanisms and outcomes occur together.

Realist evaluations can utilize a variety of different methods in order to develop the initial program theory [10]. These methods can include literature review, document review, or interviews or focus groups with organization members. Additionally, the methods used to develop the program theory are usually different from the methods used to test and refine it [10]. Therefore, the current study involved two phases, encompassing four steps: Phase 1 included (1) initial theory development and (2) initial theory refinement; Phase 2 included (3) theory testing and (4) refinement of the program theory.

## Method

### Phase 1

Phase 1 involved a qualitative realist evaluation of Progress Place. The evaluation involved a participatory approach, which included involving the community partner at each stage of the project: recruitment, data collection tool development, and interpretation. Drafts of the analyses and reports were presented to the staff and members in order to allow for feedback, refinement, and edits. The finalized demi-regularities were presented to staff and members in order to validate that they represented the experiences of members. A more detailed description of the participatory approach utilized in Phase 1 has been documented elsewhere [8]. Focus groups were conducted to uncover Context-Mechanism-Outcome (CMO) configurations that exist for members as they utilize Progress Place. The phase one evaluation conformed to the code of ethics Guidelines for Ethical Conduct proposed by the Canadian Evaluation Society [11].

### Sample

Thirty-nine individuals from three groups (members, staff, and board of directors) participated in six focus groups. Most groups involved a mixture of both members and staff, which is consistent with the Clubhouse standards of promoting collegial relationships. Participants were not asked whether they are staff or members, as the Clubhouse does not acknowledge the difference between staff and member as meaningful. One focus group was with the members of the board of directors separately, which includes members of the public as well as a number of active Clubhouse members. Demographic information was not collected in order to respect the privacy of all participants. Members represented all units within the program, as well as evening and weekend users.

### Qualitative guides

#### Focus group questions

A set of six questions were asked, including a description of Progress Place, activities members engaged in at Progress Place, how programs helped members achieve outcomes, who was a good fit to participate at Progress Place, and how Progress Place differs from other organizations (see Additional file 1).

### Procedure

For step 1, Progress Place staff and the Executive Director asked members if they would like to participate in the evaluation. The Executive Director informed the board of directors of the evaluation. The Clubhouse staff were also invited to participate in the evaluation. Two members of the research team conducted the focus groups in person and over the phone (board of directors group only). Step 2 involved confirming the themes uncovered in Step 1 via additional interviews (*N* = 4) with staff and members. The theory was then further refined by researchers and the executive Director of Progress Place.

### Coding and interpretation

Focus group transcripts were coded using Braun and Clarke’s guidelines [12] with a goal towards developing a realist theory of how and why the program achieves outcomes for members. For step 1, focus groups were audio-recorded, transcribed, and coded. Based on Braun and Clarke, the research team used an inductive approach in that themes were derived directly from participant’s responses themselves. When it came time to develop the realist theory, the themes were organized in a framework consistent with the CMO approach used in realist methodology. However, it was not a theoretical thematic analysis in that no existing theory was imposed on or sought after while analyzing the data. In keeping with the inductive approach, participants’ words, language, and quotes were retained as much as possible in the naming of the themes. Consistent with an essentialist/realist thematic analysis, motivations, experiences, and meanings were theorized in a straightforward way based on language participants used in the interviews. As the goal was to describe a theory from a realist perspective, meanings and themes were examined across the whole data set. For step 2, the researchers, alongside the program Executive Director, refined the theory by reducing or eliminating redundancy in the mechanisms and outcomes.

### Phase 2

Phase 2 involved a quantitative examination of the realist theory created in phase 1. The mechanism-outcome demi-regularities identified by Phase 1 offer Progress Place a theory to explain how their members benefit from the Clubhouse. From the realist perspective, single evaluations cannot produce universally valid findings [13]. Therefore, the purpose of the second phase of the evaluation was to test, validate, and refine the pre-existing theory of psychosocial rehabilitation at Progress Place using quantitative data. Phase two of the evaluation gained ethical approval through Ryerson University’s Research Ethics Board (REB # 2016–178), which handled the concerns of research in a vulnerable population.

### Measures

Participants were asked to participate in an online or paper-based questionnaire that assessed demographic information as well as the constructs identified as mechanisms and outcomes from Phase 1. Mechanisms and outcomes were examined using visual analogue scales (VAS). The VAS scales asked members to rate on a scale from 0 to 100, using a visual spectrum, the degrees to which they felt on a specific variable. These scales were developed with language used by members in the previous evaluation [8]. Therefore, these scales reflect the constructs to be measured and are consistent with how the constructs were described by members.

#### Demographics, housing, vocational, and service use history (DHHS)

The demographic questionnaire inquired about age, sex, education level, employment status, income, ethnicity, cultural background, family composition, and presence of disability or mental health illness [14]. The demographic items that were chosen for the present study are those that, in collaboration with Progress Place, were found to be relevant.

#### Visual analogue scales (VAS)

Visual analogue scales were used to measure mechanisms and outcomes with the language that was used in by participants Phase 1. VAS are psychometric response scales used for subjective variables that are unable to be measured by existing questionnaires. In VAS, individuals respond by specifying their level of agreement with a statement by choosing a particular point along a continuum. In the present study, participants rated on a visual scale from 0 to 100 how much they agreed with the given statement. The following phrases represent a number of questions that were asked using VAS:

- Sense of connection and belonging: Please mark on a scale of 0–100 the extent you feel a sense of belonging and social connectedness at Progress Place where you can fully be yourself.
- Dignity and self-worth: Please mark on a scale of 0–100 the extent you feel that you have value and worth as a person in society regardless of your mental health status.
- Skills acquired: Please mark on a scale of 0–100 the extent you feel that you have gained knowledge and skills at Progress Place that you can use in a volunteer or paid work experiences.

### Procedure

The questionnaire was made available to participants online using Qualtrics and through a paper-based version, both of which were accessed on-site at Progress Place. The second phase of the evaluation also involved a participatory approach in that members and staff were involved in all aspects of the research including creating the measures, assisting in data collection, providing feedback on results, and presentation of the findings. A comprehensive discussion of the participatory approach of the present study can be found elsewhere [15]. The entire questionnaire took approximately 15 to 20 min to complete.

### Statistical analysis

Hierarchical regression analyses were chosen in order to examine whether mechanisms were significantly associated with outcomes above and beyond demographic information. Each outcome was entered as the dependent variable in the hierarchical regression analysis, making three separate regression analyses. Demographic information (gender, age, education) and service utilization (frequency and length of involvement) were entered in block 1 of the analysis and the corresponding mechanisms for each outcome from Phase 1 were entered into block 2. These analyses were done in order to test whether the mechanism variables were significantly associated with the development of outcomes beyond the effect of demographic information.

## Results

### Phase 1

The objectives of Phase 1 were to create a theoretical model using a realist perspective to identify how, for whom, and under what circumstances Progress Place achieved outcomes, as well as to validate this model with stakeholders (members and staff). In the realist approach, the overall model can be considered as a set of Context-Mechanism-Outcome Configurations. When a full set of CMO configurations does not exist within the data, but instead, patterns of Context-Mechanism, Mechanism-Outcome, or Context-Outcome exist, the term demi-regularities are used to describe the patterns [16].

### Context

Initially, a set of questions was used to explore the contextual differences that might impact mechanism-outcome associations. In keeping with the research, contextual differences were identified as characteristics about the setting, the service recipients, and those delivering the program. Interestingly, participants were unable to articulate the contextual piece of Progress Place that is standard within a realist evaluation. Rather, participants stated that Progress Place could work for anyone, no matter their background or stage of recovery.

You know, somebody comes in and is at a certain stage of their emotional well-being… the friendships that they build, the activities that they participate in. You know, progress that is done slowly. And I think Progress Place allows for that growth… Not everybody who walks in the door would be qualified, for example, to participate in the employment program. I think it might take a while. So, I think there are different phases that they can get to and then, you know, get wings and fly off… So yes, I think that Progress Place does allow for, for a reasonably large spectrum of where you’re at with mental illness.-Focus group 3.

Attempts were made to reword questions and examples in order to further explore contextual variables, but subsequent participant responses did not yield additional information. Participants, regardless of whether they were members, staff, or members of the board of directors, repeatedly discussed that given the solid foundation of the program through its Quality Standards, and the flexibility of staff, that anyone will benefit from the program.

### Mechanisms and outcomes of recovery

**Fig. 1:**
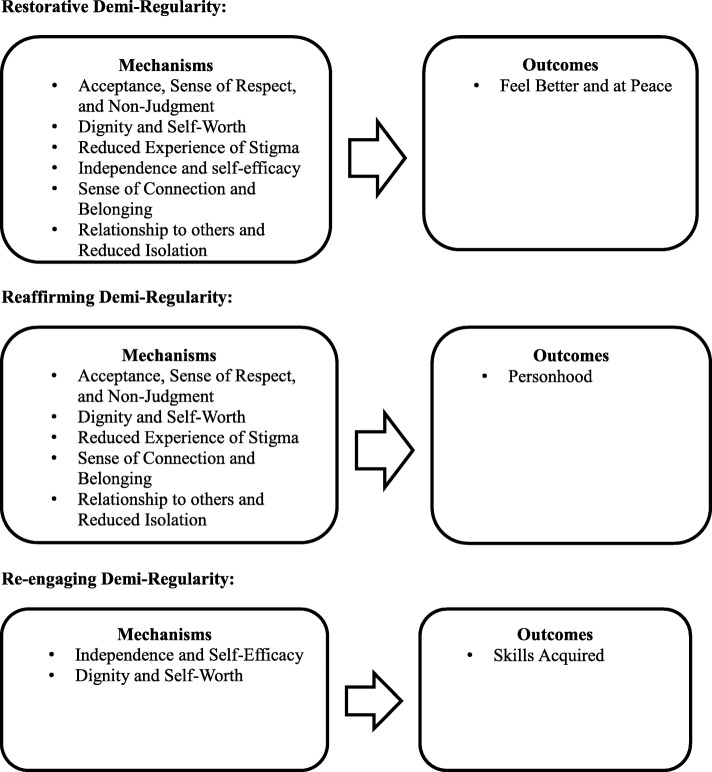
Phase 1 Demi-Regularities

From the analysis of the focus groups, three Mechanism-Outcome demi-regularities are proposed (see Fig. 1). The restorative demi-regularity involves the development of the outcome: feeling better and at peace (O_1_). The outcome was achieved by the development of the following subjective mechanisms: acceptance, sense of respect and non-judgment (M_1_); dignity and self-worth (M_2_); reduced experience of stigma (M_3_); independence and self-efficacy (M_4_); relationship to others and reduced isolation (M_5_); and a sense of connection and belonging (M_6_). The reaffirming demi-regularity involves the development of a feeling of personhood (O_2_). This outcome was achieved by the development of the mechanisms: acceptance, sense of respect and non-judgment (M_1_); dignity and self-worth (M_2_); reduced experience of stigma (M_3_); relationship to others and reduced isolation (M_5)_); and sense of connection and belonging (M_6_). The re-engaging demi-regularity involves acquiring skills (O_3_), which was developed through the mechanisms of independence and self-efficacy (M_4_); and dignity and self-worth (M_2_).

### Restorative Demi-regularity

The restorative demi-regularity involves the achievement of the outcome feeling better and at peace, which was described as feeling at peace with their symptoms and themselves as a whole.

Like, I remember at one point, like going into a shopping center or going to do little things was really hard, like I, I’d hear things, I’d see things and it was hard… But now I can go places alone, I can do everything on my own. And, like I can depend on me and like, I don’t feel weird anywhere. Now I tell myself, [name] don’t worry, sometimes these things happen, it’s only in your head, you know? Like, you just go out there and you do what you have to do and you feel great after accomplishing everything… And it took me a while to feel that confidence, but I’m getting there. - Focus group 1.

This participant discussed their progression in the restorative demi-regularity. The individual stated that by attending Progress Place they have become at peace with their mental health symptoms and themselves (O_1_). This outcome was achieved through independence and self-efficacy (M_4_), highlighted in this quote as the individual discussed their ability to do things on their own. Additionally, dignity and self-worth (M_2_) is discussed in this quote as the individual reflects on depending on themselves and achieving their goals. Lastly, the mechanism, reduced experience of stigma (M_3_), is highlighted throughout as the individual now feels comfortable going out and accomplishing their goals, regardless of their mental health symptoms.

We use language like membership which is a, conveys a sense of belonging. And we set up our work so that there is a mutual, reciprocal need for each other. That, and that it is accepting of basically anybody who wants to be a member. Because we have that kind of community, sense of belonging… I say that we help people with correcting Maslow’s hierarchy of needs in our life. Where, they may have their safety and their security, but their sense of community and belonging has been reversed… So, we try to correct…we can create this intentional community where people do have a sense of belonging and being needed and being wanted and expected. And therefore, they regain their esteem so that they can go out and venture out and achieve. - Focus group 4.

The participant discussed the mechanisms: acceptance, sense of respect and non-judgment (M_1_) by stating that anyone can become a member and they will be accepted into the Progress Place community. Secondly, the participant stated that Progress Place promotes relationship to others and reduced isolation (M_5_); and a sense of connection and belonging (M_6_). This participant also stated that individuals regain their dignity and self-worth (M_2_) as well as independence and self-efficacy (M_4_) in achieving their goals. These mechanisms all engender the development of feeling better and at peace (O_1_).

### Reaffirming Demi-regularity

The reaffirming demi-regularity involved the development of a feeling of personhood. Participants in the study described personhood as being viewed as a person beyond their mental health symptoms.

At Progress Place you don’t look at a person as a case, you don’t look at a person as their illness. We don’t, we look at a person as a person. We deal with [them] as a person and that’s what [member’s name] says, you know that relationships are very important for us. And that’s one thing I think is very important, at Progress Place… You don’t look at a diagnosis, you look at a person. You know. And you build a relationship, and you know the person by what they are and then, you know, working together. - Focus group 4.

You choose what’s right for you. And I think when people make choices, you know look, like when things are, say going well and they are making choices, they’re taking on responsibilities and they’re reminding themselves on a, like on a daily basis that I’m strong enough that I can take on doing tours. And I’m strong enough that I can, you know, answer the switchboard. Or, you know I’m not as fragile as I may have been when I was ill. Like, this is, I’m on the recovery side or it’s reinforcing of that on a daily basis. And it moves people away from like, like you, like someone mentioned that they have a voice. That I have choices. That I’m not in patienthood, I’m moving into personhood. - Focus group 4.

These quotations reflect the mechanism-outcome configuration of the reaffirming demi-regularity. Personhood is achieved by feeling acceptance, sense of respect and non-judgment (M_1_) discussed in both quotations as accepting a person regardless of their mental health status. Secondly, dignity and self-worth (M_2_) is reflected in the second quotation as being able to take on responsibilities and not feeling fragile. The first quotation reflects the importance of relationships with others and reduced isolation (M_5)_); and sense of connection and belonging (M_6_) as individuals work together at Progress Place to achieve the outcome of personhood (O_2_).

It’s a community of people that are, share a common, a common struggle in dealing with mental illness, mental health issues, and do collective work and individual support. They’re able to regain as [other participant] said, their confidence. They can regain aspects of their life to get back to, you know, living a full life, making a, making contributions to society, which drives everybody. But, they do have, the common theme they all have an experience with mental illness and that’s the common thread. But, after that it becomes just a group of people trying to help each other. - Focus group.

This participant, a member of the board of directors, is discussing the development of personhood through the mechanisms: acceptance, sense of respect and non-judgment (M_1_) in that all members have a common struggle in dealing with mental illness. Members regain dignity and self-worth (M_2_), which is reflected by this member as regaining their confidence. They have a reduced experience of stigma (M_3_) and increased independence and self-efficacy (M_4_), because members are able to participate in collective work and regain aspects of their life. Lastly, the common experience between the members promotes relationships with others and reduced isolation (M_5_) and a sense of connection and belonging (M_6_), leading to feeling like a person beyond their mental illness (O_2_).

### Re-engaging Demi-regularity

The final configuration is the re-engaging demi-regularity, which involved the outcome of acquiring skills; defined by participants as social skills, work related skills, and daily life skills.

I like to become very independent and know how to do everything, but if there’s something I don’t know how to do, there’s a lot, the staff here, four of them are here, and they very much, they’ll help you out and get you on the straight and narrow as well as if something’s broken down, “Help! Help! Help!” you know [laughs]… But at the same time, I like to feel independent doing my work and not always having to ask all the time. I like to get to know what I’m doing and do it well and not having to ask for help every single minute of the day. - Focus group 4.

Everyone and anyone who struggles or has a mental health issue can benefit very much from coming here. Very much because there’s just so much you can do here and you, you get, you feel a lot of hope seeing, seeing yourself work and ah, like work, complete tasks and be successful. And it always builds on your confidence level and your self-esteem eventually gets higher because you know that you’re acquiring more and more every day when you’re doing new things, learning new skills or practicing the skills you already have. - Focus group 5.

These two quotations reflect the outcome of acquiring skills, which is developed by the mechanisms independence and self-efficacy (M_4_); and dignity and self-worth (M_7_). The members discussed how they like to feel independent in their work (M_4_), and while sometimes they need to ask for help, being independent builds their dignity and self-worth (M_7_). The second quote shows the progression of building confidence and self-esteem, and how that leads to acquiring new skills or practicing skills they have already gained (O_3_).

### Phase 2

**Fig. 2:**
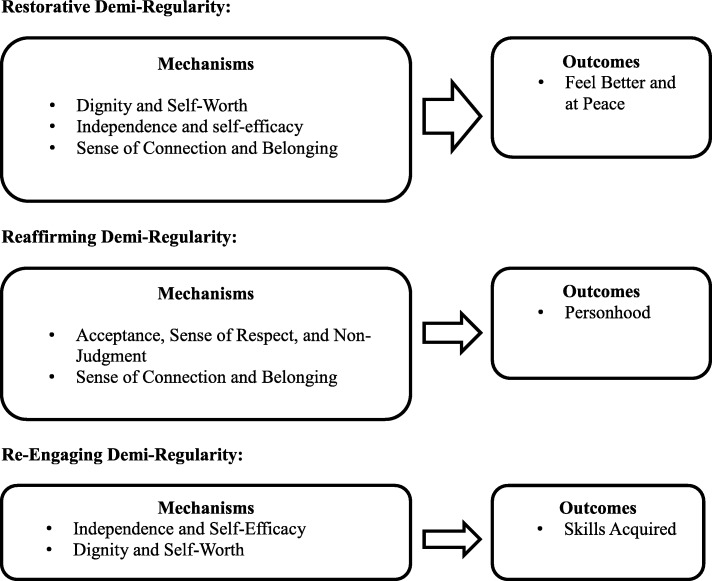
Phase 2 Demi-Regularities

Descriptive statistics of the VAS measures can be found in Table 1. Multicollinearity statistics for the hierarchical regression analyses are below the standard cutoff of the VIF being less than ten [17], suggesting that multicollinearity did not exist in the analyses (see Table 2). The results of the hierarchical regression models remained consistent with those found in phase 1 (see Fig. 2).

**Table 1:** *Descriptive Statistics of Mechanisms and Outcomes*

	VAS Measure^a^	M	SD	95% Confidence Interval	Response Range
Mechanism	Acceptance, equality, and non-judgment	80.1	20.7	[76.83, 83.34]	0–100
	Dignity and self-worth	77.3	21.5	[73.88, 80.65]	1–100
	Reduced experience of stigma	83	20.1	[79.78, 86.15]	10–100
	Independence and self-efficacy	81	19.8	[77.79, 84.04]	8–100
	Relationship to others and reduced isolation	74	23.8	[70.02, 77.63]	0–100
	Sense of connection and belonging	79	21.9	[75.20, 82.24]	0–100
Outcome					
	Feeling better and at peace	74	23	[70.31, 77.55]	0–100
	Personhood	83	18.9	[80.00, 85.93]	0–100
	Skills Acquired	75	23.6	[71.53, 79.05]	0–100

^a^VAS scale values ranged from 0 to 100

**Table 2:** *Multicolinearity Statistics*

Outcome	Mechanism	B	SE	Tolerance	VIF
Feeling better and at peace					
Acceptance		.070	.095	.569	1.758
Dignity		.353	.090	.530	1.886
Reduced stigma		−.103	.102	.471	2.123
Efficacy		.288	.095	.554	1.806
Isolation		−.004	.079	.570	1.753
Belonging		.273	.103	.401	2.495
Personhood					
Acceptance		.230	.073	.649	1.540
Dignity		.089	.073	.593	1.686
Reduced stigma		.099	.086	.498	2.010
Isolation		.040	.067	.588	1.700
Belonging		.289	.085	.430	2.325
Skills acquired					
Dignity		.266	.094	.695	1.438
Efficacy		.448	.102	.695	1.438

### Sample

The goal for Phase 2 was to recruit approximately 100 members at Progress Place who are currently active, meaning they use the Clubhouse regularly, representing approximately 15% of the total population of users at Progress Place. There were 168 members who participated in Phase 2; ten were excluded from analysis due to being incomplete. Of the remaining participants, the sample included 43.7% female respondents, with a mean age of 48 (ranging from 17 to 75 years). With respect to mental health status, 36.7% of members indicated that they have depression, 29.1% anxiety, 36.1% schizophrenia, 22.2% bipolar, and 2% reported other mental illnesses (i.e., borderline personality disorder).

As well, 24.1% reported that they have been members for less than one year, 34.8% for one to five years, 18.4% for five to eight years, and 22.8% have been members for more than 10 years. With respect to frequency of visits, 23.4% of members stated they attended Progress Place everyday, 69% stated they come in at least once a week, and 7% reported use every couple of weeks.

### Restorative Demi-regularity

The results of Phase 1 suggested that feeling better and at peace would be predicted by the mechanisms: acceptance, sense of respect, and non-judgment (M_1_); dignity and self-worth (M_2_); reduced experience of stigma (M_3_); independence and self-efficacy (M_4_); relationship to others and reduced isolation (M_5_); and sense of connection and belonging (M_6_). In Phase 2, containing all demographic information and mechanisms, independence and self-efficacy (*p* = 0.022), dignity and self-worth (*p* < 0.000), and sense of connection and belonging (*p* = 0.006) were significantly associated with the subjective outcome feeling better and at peace (see Table 3).

**Table 3:** *Model Summary for Feeling Better and at Peace*

	Variable	B	Std. Error	Beta	t	R^2^	R^2^ Change
Model 1	(Constant)	64.499	15.962		4.041	.061	.061
	Gender	−3.505	3.539	−.089	−.990		
	Education	2.503	1.767	.126	1.416		
	Age	−.314	.178	−.172	−1.765		
	Length	1.720	1.160	.145	1.482		
	Frequency	3.338	2.517	.119	1.326		
Model 2	(Constant)	2.386	14.287		.167	.516	.456***
	Gender	−4.240	2.659	−.107	− 1.594		
	Education	1.263	1.371	.064	.921		
	Age	−.108	.135	−.059	−.800		
	Length	.222	.906	.019	.244		
	Frequency	1.745	1.885	.062	.926		
	Acceptance	.114	.098	.103	1.160		
	Dignity***	.364	.091	.348	4.006		
	Stigma	−.147	.120	−.134	−1.223		
	Efficacy*	.225	.097	.197	2.316		
	Belonging**	.330	.117	.311	2.819		
	Isolation	.004	.079	.004	.049		

*Significant at *p* < 0.05

**Significant at *p* < 0.01

***Significant at *p* < 0.001

### Reaffirming Demi-regularity

The results of Phase 1 showed that personhood would be predicted by the mechanisms: acceptance, sense of respect, and non-judgment (M_1_); dignity and self-worth (M_2_); reduced experience of stigma (M_3_); sense of connection and belonging (M_6_); and relationship to others and reduced isolation (M_5_). In Phase 2, the model found that the mechanisms, acceptance, sense of respect, and non-judgment (*p* < 0.000), and sense of connection and belonging (*p* < 0.002), were significantly associated with the subjective outcome of personhood (see Table 4).

**Table 4:** *Model Summary for Personhood*

	Variable	B	Std. Error	Beta	t	R^2^	R^2^ Change
Model 1	(Constant)	104.701	13.608		7.694	.064	.064
	Gender	−1.469	3.021	−.043	−.486		
	Education	.811	1.507	.048	.539		
	Age*	−.365	.152	−.233	−2.406		
	Length	.010	.986	.001	.010		
	Frequency	−1.597	2.152	−.066	−.742		
Model 2	(Constant)	41.801	12.000		3.483	.511	.448***
	Gender	−2.386	2.268	−.071	−1.052		
	Education	.282	1.157	.017	.244		
	Age	−.243	.115	−.155	−2.116		
	Length	−.216	.768	−.021	−.282		
	Frequency	−1.434	1.614	−.060	−.888		
	Accept***	.266	.074	.294	3.606		
	Dignity	.109	.075	.122	1.462		
	Stigma	.064	.101	.068	.634		
	Belonging**	.304	.097	.334	3.142		
	Isolation	.027	.067	.034	.409		

*Significant at *p* < 0.05

**Significant at *p* < 0.01

***Significant at *p* < 0.001

### Re-engaging Demi-regularity

Lastly, the third subjective outcome that was examined was skills acquired. Phase 1 found that this outcome would be predicted by the mechanisms independence and self-efficacy (M_4_); and dignity and self-worth (M_2_). In Phase 2, containing all demographic information and mechanisms, both variables were found to be significant (self-efficacy, *p* < 0.000; dignity and self-worth, *p* = 0.005; see Table 5).

**Table 5:** *Model Summary for Skills Acquired*

	Variable	B	Std. Error	Beta	t	R^2^	R^2^ Change
Model 1	(Constant)	69.456	16.584		4.188	.049	.049
	Gender	−1.125	3.733	−.027	−.301		
	Education	1.829	1.852	.087	.987		
	Age	−.403	.187	−.208	−2.156		
	Length	1.619	1.215	.129	1.332		
	Frequency	3.274	2.580	.113	1.269		
Model 2	(Constant)	22.503	16.131		1.395	.290	.242***
	Gender	−.884	3.254	−.021	−.272		
	Education	−.156	1.641	−.007	−.095		
	Age	−.186	.166	−.096	−1.120		
	Length	.574	1.072	.046	.536		
	Frequency	2.020	2.257	.070	.895		
	Dignity*	.203	.101	.182	2.000		
	Efficacy***	.472	.111	.386	4.231		

*Significant at *p* < 0.05

**Significant at *p* < 0.01

***Significant at *p* < 0.001

## Discussion

The purpose of the present study was to test, refine, and validate a realist theory of psychosocial rehabilitation at Progress Place. The present theory began with the principles of psychosocial rehabilitation, which, alongside qualitative data from a realist evaluation, informed the creation of three Mechanism-Outcome demi-regularities. The second phase of the evaluation tested and refined the realist program theory. The three mechanism-outcome demi-regularities represent a holistic model of recovery for members as they utilize Progress Place. The results support the notions by Deegan who described that service users are not passive recipients of services, and rather, recovery involves developing a new sense of self and purpose beyond a mental illness [18]. Psychosocial rehabilitation offers the services to support individuals in their recovery process, including the psychological changes of accepting and overcoming the challenges of mental illness [18].

### Consistency with previous outcome studies

The present evaluation indicated that the outcome feeling better and at peace is an important aspect of recovery for members at Progress Place. A study by Hancock and colleagues interviewed Clubhouse members who scored high on the Recovery Assessment Scale in order to identify factors of the later stages in recovery [19]. One outcome that was identified was accepting the illness and gaining control over symptoms. This outcome parallels feeling better and at peace in the present study: feeling better, in that Clubhouse members gain control over their symptoms, as well as being at peace with your mental illness, such as accepting that symptoms will not disappear completely.

Personhood has been well documented in the psychosocial rehabilitation literature as an outcome of recovery that involves the remaking of a sense of being a person outside of the mental illness diagnosis [20, 21]. Personhood has previously been discussed as the principle that transcends all others when working with people with severe mental illness [21] and is one of the key factors of recovery when evaluating the quality of mental health care [22] Results from the realist evaluation identified mechanisms that lead to the development of a sense of personhood, including sense of connection and belonging, as well as acceptance, sense of respect, and non-judgment. A recent study reported that Clubhouse members do not feel judged, and rather feel accepted and understood for who they are promotes having “human value” for Clubhouse members [20], paralleling the Reaffirming demi-regularity that was described in the present study.

Acquiring skills was found to be an outcome of recovery for members as they utilize Progress Place. Psychosocial rehabilitation programs believe that skill development will assist individuals through their recovery by enabling them to live a more typical lifestyle [1]. Hancock and colleagues [19] found that individuals in the later stages of recovery discuss contributing through meaningful activity is an important recovery outcome. Specifically, they discuss that gaining skills for volunteer or paid employment, or doing other purposeful activity, is a key factor in recovery.

The present evaluation found a number of mechanisms that were significantly associated with outcomes, which have also been documented previously in the psychosocial rehabilitation literature. A study by Arns and Linney [23] found that the development of self-esteem in a psychosocial rehabilitation program predicted the development of life satisfaction. A more recent study has found that participating in the activities at the Clubhouse, i.e., the work-ordered day, leads to the development of dignity and self-worth [20]. Research on Clubhouses has found that individuals with severe mental illness who have larger or more satisfactory social networks report higher quality of life than those who do not [24].

The second phase of the evaluation found that a sense of connection and belonging was significantly associated with the outcome of feeling better and at peace and the outcome of personhood. Conversely, relationship to others and reduced isolation was not a significantly associated with outcomes in Phase 2. Previous research has indicated that features of the social network such as the extent of the support and the reciprocal nature of the relationship influence a subjective sense of recovery [25]. Additionally, Conrad-Garrisi and Pernice-Duca [26] found that a sense of mattering, described as being attended to, being concerned about, and regarded as significant, were predictive of a subjective sense of recovery. These studies suggest that it is not quantity of the social relationship, rather the quality that affects recovery outcomes. Members of Progress Place also stated that they have more independence and self-efficacy in making their own choices and have the skills in order to make plans, problem solve, and act independently. Self-efficacy has been found to increase feelings of recovery and personhood through members’ newly acquired skills [27].

### Complexity of the clubhouse model

It is evident that the majority of the previous literature on Clubhouses has focused on functional outcomes such as employment, skill development, and social networks, with little attention given to the psychological processes that change for members as they engage in Clubhouses. In addition, researchers have assumed what the outcomes from Clubhouses should be and have not attempted to extract an understanding from member’s perspectives. Understanding and describing the program theory is vital in order to understand how psychosocial rehabilitation is impacting members. The current study has provided an understanding of the changes and outcomes that occur for members, from the perspective of the key stakeholders in the Clubhouse. This information has provided insight into the complexity of the inter-connections of the demi-regularities within the Clubhouse setting and shows that providing a wide range of programs tailored to all levels of functioning leads to many mechanisms and outcomes. The research has implications for other community mental health providers and suggests that recovery, from the perspective of service recipients, is a complex process not merely concerned with functional outcomes.

This type of in-depth qualitative work requires trust between the research and community partners, who are likely looking for functional data that can provide future funding opportunities. The realist perspective requires the community organization and academic partner to push pause on collecting data about functional outcomes, in order to get a better understanding of how outcomes are achieved. Therefore, the realist perspective is often difficult to achieve in community-based organizations unless a longstanding, trusting relationship has already been developed. Due to the collaborative nature of the present project, a realist theory of Progress Place was created and offers significant insight into the processes that occur for individuals who access psychosocial rehabilitation programs.

### Limitations

There are a number of limitations to be acknowledged in the present study. One limitation includes the limited data obtained on contextual factors. Interestingly, participants reported that the organization would work for anyone, regardless of their mental health status or level of engagement. It was difficult for members and staff to articulate the context because there are so many choices and options that members can engage with based on their current preferences and priorities. The Quality Standards for Clubhouses states, “All members have equal access to every Clubhouse opportunity with no differentiation based on diagnosis or level of functioning” [2]. As member preferences for involvement expand or become more limited, the options for engagement in various programming can change. Contextual variables within the Clubhouse program are an area that requires further exploration.

The sample that was obtained in the evaluation consisted of high frequency users of Progress Place. Additionally, no comparison sample was used to measure differences between the mechanisms and outcomes for individuals who attend Progress Place and those who do not. In place of a control group, it is recommended that Progress Place continue collecting data on these mechanisms and outcomes in order to track members over time. This data would provide information about whether members at Progress Place change over time, and would also suggest that, regardless of severity, individuals are experiencing changes in their outcomes. The theory that was created at Progress Place may not be generalizable to other psychosocial rehabilitation programs. Future research should evaluate whether the realist theory of psychosocial rehabilitation developed and refined in the present evaluation remains stable across Clubhouses. Lastly, the positivist methods utilized in the present study are limited in that they offer only one explanation of reality that is occurring for members of the Clubhouse. However, realist methodologists have suggested that utilizing both qualitative and quantitative methods allows for different facets of the same reality to be revealed and can be used to examine reality from different perspectives [28]. Therefore, the present study is limited, but provides a detailed explanation of reality in line with the critical realist framework.

## Conclusions

The current evaluation has developed, tested, and refined a realist theory of psychosocial rehabilitation at Progress Place, represented by three mechanism—outcome demi-regularities. The realist theory indicates that there are various mechanisms that produce outcomes of recovery from participating at Progress Place, and it is necessary that community-based programs promote a multifaceted, holistic perspective on recovery. Although housing has been found to increase individual’s quality of life [29], the present study suggests that several additional factors other than housing may be involved in successful recovery. Progress Place and other Clubhouses that offer programming in addition to housing, may be able to produce many more recovery mechanisms and outcomes for members. In conclusion, the present realist evaluation found that Progress Place is providing a program that is supporting recovery for its members. Due to this two-phase evaluation, a refined realist theory of psychosocial rehabilitation, supported by the literature, has been created.

## Additional file

Additional file 1: Focus Group Discussion Guide. The interview discussed guide attached as an additional file was used to direct the discussions of both the group interviews and focus groups. (DOCX 67 kb)
