# Supplementary material for: Developing a realist theory of psychosocial rehabilitation: the Clubhouse model
Source: BMC Health Serv Res. 2018 Jun 13;18:442. doi: 10.1186/s12913-018-3265-9 (PMC6004683; doi:10.1186/s12913-018-3265-9)
Supplement: Supplementary file 1 — Focus Group Discussion Guide. The interview discussed guide attached as an additional file was used to direct the discussions of both the group interviews and focus groups. (DOCX 67 kb) [file 12913_2018_3265_MOESM1_ESM.docx]

**Focus Group Questions for Members and Staff**

The purpose of this group is to understand how Progress Place helps those living with mental health issues.

1. Thinking about the different places, services, and groups within Progress Place, which ones have you joined?
2. Thinking about the different areas of your life you might need help with, which ones has Progress Place helped you with?
3. If you had to describe to someone who has never been to Progress Place, how would you explain what Progress Place does?
4. Thinking about other people in the community who have mental health issues, who would be a good fit with Progress Place?
   1. For example; different groups of people: youth, older adults, men and women.
   2. Who would be a better fit somewhere else?
5. There might be other groups, agencies, or professionals in the community who also support you. What groups are they?
6. Thinking about Progress Place, how is different than those groups?
   1. For example, it could help with DIFFERENT areas or parts of your life (describe)
   2. For example, it could help in a DIFFEERENT way (describe)
